# Outcomes with heart failure management in a multidisciplinary clinic - A randomized controlled trial

**DOI:** 10.1016/j.ihj.2022.06.005

**Published:** 2022-06-14

**Authors:** Bhagwati Prasad Pant, Santhosh Satheesh, Ajith Ananthakrishna Pillai, Avinash Anantharaj, Lakshmi Ramamoorthy, Raja Selvaraj

**Affiliations:** a Department of Cardiology, Jawaharlal Institute of Postgraduate Medical Education and Research, Pondicherry, India; b Department of Nursing, Jawaharlal Institute of Postgraduate Medical Education and Research, Pondicherry, India

**Keywords:** MDHFC, Multidisciplinary heart failure clinic, QoL, Quality of Life, HFrEF, Heart failure with reduced ejection fraction

## 1 Introduction

HF is a complex clinical syndrome, current global health priority and a leading cause of hospitalization.^1^ Nearly 25% of patients with HF are re-hospitalized within a month and 50% within 6 months of discharge.^2^ Disease management through MDHFC has been shown to reduce re-hospitalization, improve QoL and reduce mortality in the western population.^3^ There is no data available regarding the feasibility of setting up a MDHFC and outcomes in an Indian population.

## 2 Materials & methods

This was a parallel group, Randomized control, single centre study. Participants comprised of adult patients (>18 years) with stable HFrEF. Patients with recent MI or coronary revascularization were excluded. The patients underwent block randomization. Patients in the intervention group were followed up in the MDHFC. Patients in the control group were followed up in the cardiology outpatient clinic (usual care) from July 2019 to August 2020.

### 2.1 Multidisciplinary heart failure clinic

Intervention consisted of follow up in a specialized HF clinic. It comprised of the treating cardiologist, trained nurses, social worker, dietitian and other specialties on demand. The key features of interventions were clinical assessment, timely optimization of HF medications till the achievement of target doses at every visit by treating cardiologist.

**Fig. 1:**
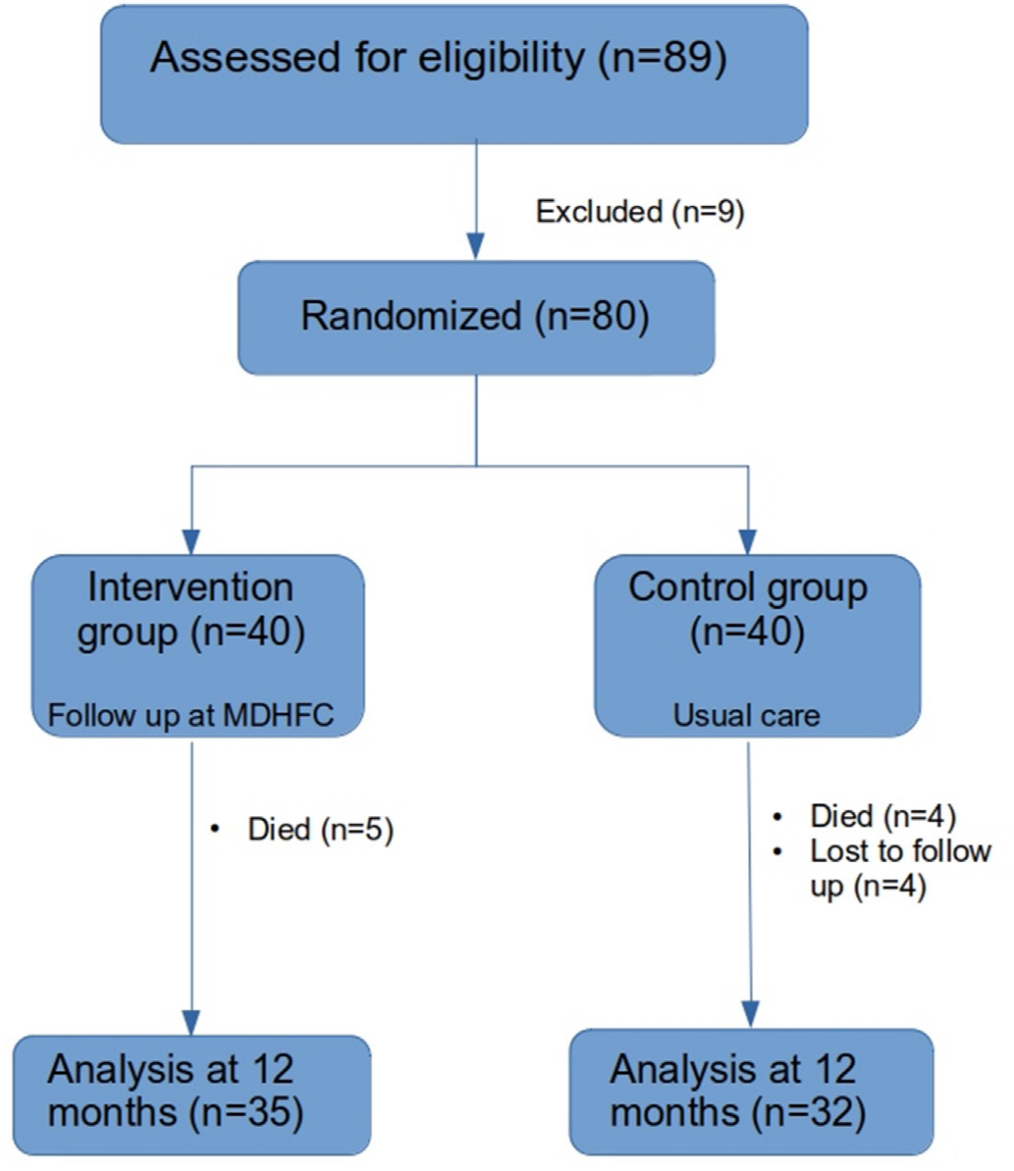
Flow chart of patient recruitment and follow-up for 1 year.

Patients in control group attended the routine outpatient cardiology clinic. Outcomes were measured at 1 year. Primary end point was a composite of death from any cause and hospitalization for HF. Secondary end points were death from any cause, hospitalization for HF, cardiovascular death, QoL, drug adherence and exercise capacity (Fig. 1.).

### 2.2 Tools

Minnesota Living with Heart Failure Questionnaire (MLHFQ) was used to assess QoL.^4^ Drug adherence was assessed by using Morisky Green Levine (MGL) drug adherence score.^5^ Six minute walk test (6 MWT) was done to evaluate the functional capacity of the patients.^6^

### 2.3 statistical analysis

Data was analysed using both descriptive and inferential statistics. Intention-to-treat principle was followed during the data collection. Survival probability between the two groups were compared with Kaplan–Meier survival curves and log-rank test was used to calculate the difference in primary end points. We obtained a sample size of 80 patients in total to provide a power of 80% to measure a 25% reduction in this outcome with 1:1 randomization.^7^ All statistical analysis was done by SPSS statistical software (Version 23.0, SPSS Inc; Chicago).

## 3 Results

There were no significant differences in important clinical characteristics between the two groups (Table 1). The patients in the intervention group attended the clinic on an average of 13.4 ± 4.8 times in one year which was more than the control group patients who attended the usual care out-patient clinic at 9.2 ± 3.6 times in one year.

**Table 1:** Baseline characteristics of patients.

Demographics	Intervention group (n = 40)	Control group (n = 40)
Age (Mean ± SD)	54.5 ± 9.6	55.6 ± 9.9
M:F	36:4	33:7
Socioeconomic status		
Lower class	35 (87.5%)	25 (62.5%)
Lower middle class	2 (5%)	8 (20%)
Middle class	3 (7.5%)	7 (17.5%)
Clinical		
NYHA III/II/I	12 (30%)/27 (67.5%)/1 (2.5%)	6 (15%)/30 (75%)/4 (10%)
Mitral regurgitation		
Nil- 2+/3–4+	33 (82.5%)/7 (17.5%)	33 (82.5%)/7 (17.5%)
Blood pressure		
SBP/DBP(Mean ± SD)	117.3 ± 20.7/75.4 ± 13.4	116.6 ± 18.3/72.3 ± 10.3
BMI(Mean ± SD)	24.8 ± 3.7	25.2 ± 2.8
Ejection fraction (Mean ± SD)	30.1 ± 4.7	31.2 ± 5.0
Atrial fibrillation	1 (2.5%)	2 (5%)
LBBB	5 (12.5%)	2 (5%)
Etiology		
Ischemic	17 (42.5%)	19 (47.5%)
Non-ischemic	23 (57.5%)	21 (52.5%)
Comorbidities		
CAD	11 (27.5%)	15 (37.5%)
HTN	15 (37.5%)	14 (35%)
DM-II	18 (45%)	12 (30%)
CKD	3 (7.5%)	4 (10%)
Biochemistry profile		
Hemoglobin (Mean ± SD)	12.5 ± 1.2	10.9 ± 2.7
S.creatinine (Mean ± SD)	1.4 ± 0.5	1.6 ± 0.1
S.Potassium (Mean ± SD)	4.2 ± 0.3	4.7 ± 0.4
Drugs		
ACEi/ARB	28 (70%)	25 (62.5%)
Beta blockers	29 (72.5%)	27 (67.5%)
Loop diuretics	38 (95%)	35 (87.5%)
MRA	26 (65%)	25 (62.5%)

**Fig. 2:**
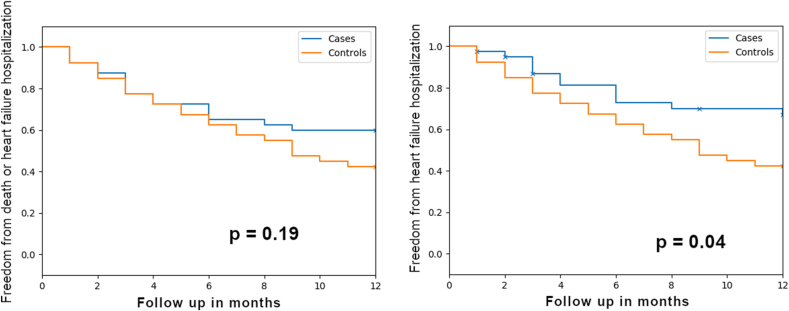
Kaplan–Meier survival estimates for Death or heart failure hospitalization (Left panel) and only heart failure hospitalization (Right Panel). The blue curve are the heart failure clinic patients while the red curve represents the control group. Follow-up was censored at 1 year.

At 1 year there were a total of 17 composite events (42.5%) in intervention group and 23 events (57.5%) in the control group (p = 0.19). Survival analysis showed no statistically significant difference in event rates. However, the curves started separating at 6 months and there was a trend towards lesser events in the intervention group (Fig. 2, panel A).

A total of 5 (12.5%) patients in intervention group and 4 patients (10%) in control group died at the end of the 1 year (p = NS). There were no non-cardiac deaths in either group. At 12 months, 24 patients (60%) in the control group, but only 12 patients (30%) in the intervention group patients had been readmitted for heart failure (p = 0.04) (Fig. 2, panel B).

Quality of Life, exercise capacity and drug adherence was better in intervention group than the control group, MLHFQ score 21.55 ± 9.0 vs 49 ± 12.6 (p < 0.01), 6 MWT 340.3 ± 55.4 mtrs vs 237.2 ± 51.8 mtrs (p < 0.01) and MGL score 0.5 ± 0.9 vs 1.8 ± 0.8 (p = 0.03) respectively.

**Fig. 3:**
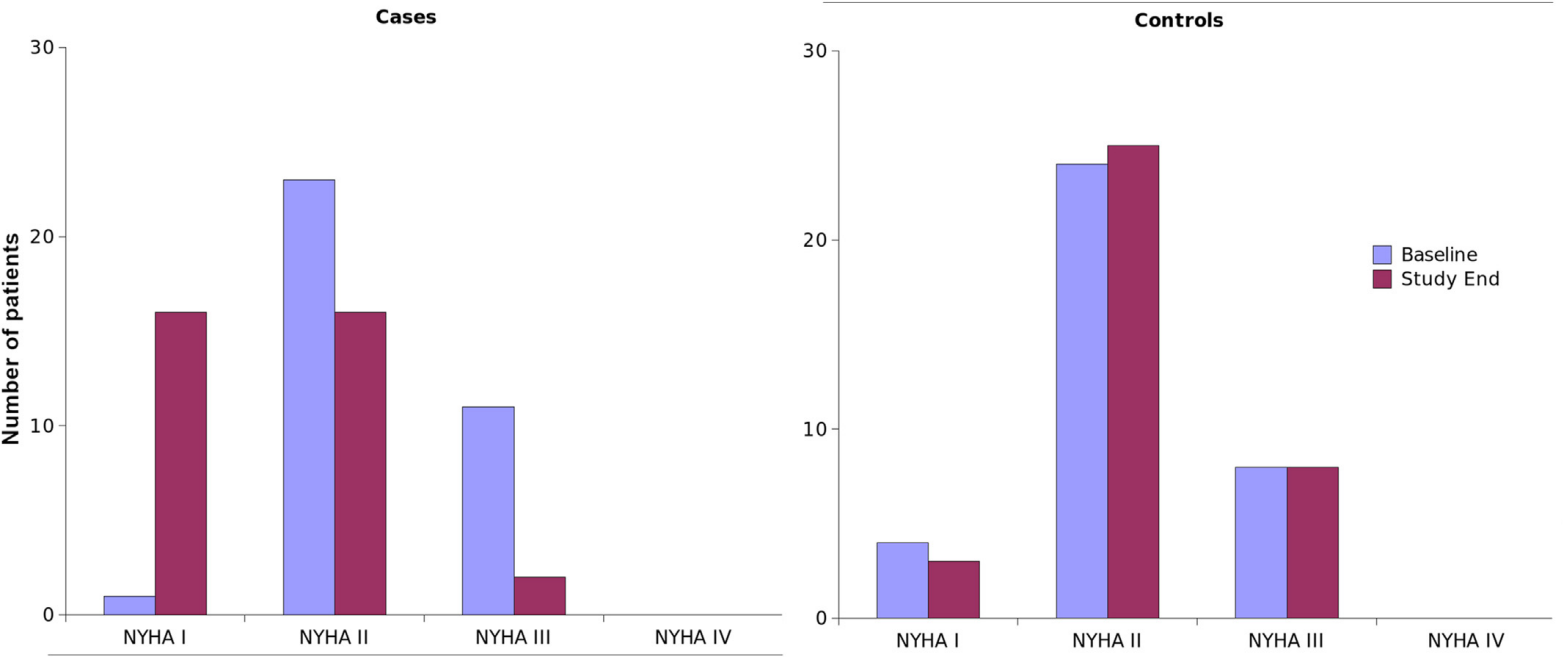
NYHA Class of cases (Left panel) and controls (Right panel) at baseline and at the end of 1 year.

Usage of ACEI/ARB, beta blockers, and MRAs was higher in intervention group (Table 2). There was significant improvement in EF in patients of intervention arm than control arm at the end of one year (Baseline-30 ± 4.8 vs 31.9 ± 3.9 at 1 year 31.5 ± 5.4 vs 28.8 ± 4.1,p = 0.001)There was improvement in the NYHA functional class of the patients in the intervention group at 1 year (Fig. 3).

**Table 2:** Medication profiles in both groups at the end of 1 year.

Drugs	Intervention group (n = 40)	Control group (n = 40)	*P* value
ACEi/ARB	33 (94%)	29 (80%)	0.015
Beta blockers	35 (100%)	28 (77%).	0.002
Loop diuretics	34 (97.1%)	33 (91%).	0.15
MRA	32 (91.4%)	20 (55%)	0.00007

## 4 Discussion

This is the first RCT in an Indian set up comparing outcomes with treatment in a MDHFC versus usual outpatient care. Our study demonstrated that follow up in a HF clinic decreased the re-admissions rates by almost 50% at 1 year. It also reiterates the benefits of multidisciplinary care in CHF patients in terms of improvement in NYHA class, exercise capacity, drug adherence and QoL. Positive trends in improvement were also observed for ejection fraction, prescription of beta blockers, ACE inhibitors, ARB and MRAs. However, like major previous randomized studies mortality benefit was not seen during the short one year follow-up.^8^

In Indian population HF strikes almost a decade early. The mean age of patients in our study was around 55 years in both the groups.^9^ Our study population was heterogenous and reflected the HF population found in Indian practice.

### 4.1 Primary end points

MDHFC care was associated with fewer composite events at 1 year follow-up. Both groups had similar number of events in initial months, however, in the later half, events in the intervention group were less. Separation of the curves after 5–6 months indicates that the benefits of multidisciplinary care starts in long term follow up and care should be continued for long term for stronger benefits. Follow up for <3 months have not shown any benefits in various studies. However studies with longer follow up designs are unlikely due to strong benefit of such clinics and frequent cross-over (Fig. 1).

### 4.2 Secondary end points

Multidisciplinary heart failure disease related programs have shown to reduce heart failure related hospitalization rates in past.^10^ In our study the predominant effect of the follow-up in a MDHF clinic was reduction in the rate of recurrent hospitalizations by almost 50% at the end of 1 year.

Reducing hospitalization is a worthwhile aim as it indirectly effects the QoL, morbidity and also health care costs.^11^^,^^12^

In chronic HF patients QoL inevitably deteriorates as the disease progresses. We found a significant improvement in the QoL scores and exercise capacity of the patients in intervention group.^13^

Drug adherence, which varies from 25 to 50% in various studies is a major problem in patients with chronic diseases.^14^ Adherence was significantly better in intervention group at the end of one year. It is likely that this improved adherence to drugs played a major role in the beneficial effects on readmission rates and QoL.

There was a considerable gap between the dosages recommended in guidelines and those given to the patients in usual care. Ten percent of the patients in the intervention group achieved target dosages of beta blockers and 42.5% of them were on more than 50% of the target dosages. This was higher than the major beta-blocker trials.^15^^,^^16^ Our study also had better proportion of patients in intervention group who were on ACEI/ARBs (94%) while 13.8% of the patients were taking target dosages and almost 63% of these patients were taking more than 50% of the target dosages at the end of one year. The CHAMP- HF registry showed only 22% of patients were treated with ACEI/ARB/ARNI, beta blockers and MRAs while <1% are on target doses of these drugs.^17^ This major finding in our study was the result of systematic implementation of the protocols for medicines to achieve maximum targeted dosages. Adherence to these drugs can drive the mortality results in our study in a long term follow up.

### 4.3 MDHFC

Components and framework of our HF clinic was on par with previous studies.^18^^,^^19^ However, our approach was unique as we emphasized one on-one comprehensive counselling sessions and also group sessions for both patients and their family members. We also identified the various obstacles faced by patients like complex medicine regimens, lifestyle modifications, rehabilitation exercise modules, dietary changes, self-care and follow up appointments etc.

## 5 Conclusions

Our framework of MDHFC demonstrated significant reduction in HF hospitalizations, improvement in QoL, functional capacity and drug adherence. There was no difference in mortality, but this is likely to be due to the short follow up period of one year. The framework of the clinic and magnitude of benefit seen in this study should help Institutes across India to decide on the need to set up such clinics.

## 6 Limitations

The low sample size in our study precludes the observation of differences in subgroups. Second, Angiotensin Receptor- Neprilysin inhibitors and Sodium-glucose cotransporter-2 were not used as they were largely unaffordable. MDHFC have shown to improve outcomes but exactly which intervention has benefited more is not known.

## Funding

None.

## Declaration of competing interest

None.
